# MV-Flow imaging for evaluating the placental function of gestational diabetes mellitus

**DOI:** 10.3389/fendo.2025.1674480

**Published:** 2025-11-25

**Authors:** Jiahao Song, Xiaobin Li, Chen Zhu, Hongshuang Sun, Yunyun Ren

**Affiliations:** 1 Department of Ultrasound, Obstetrics and Gynecology Hospital of Fudan University, Shanghai Key Lab of Reproduction and Development, Shanghai Key Lab of Female Reproductive Endocrine Related Diseases, Shanghai, China; 2 Department of General Surgery, Yangpu Hospital Affiliated to Tongji University, Shanghai, China; 3 Department of Ultrasound Medicine, Affiliated Hospital of Jining Medical College, Shandong, China

**Keywords:** microvascular flow imaging, placenta, gestational diabetes mellitus, pregnancy, ultrasound

## Abstract

**Background:**

Gestational diabetes mellitus (GDM) is characterized by vascular dysfunction and is associated with adverse perinatal outcomes for both the mother and fetus. Microvascular index (MVI) is a non-invasive marker of microvascular function assessed by microvascular flow imaging (MV-Flow). This study aimed to evaluate the performance of conventional Doppler ultrasound and MV-Flow in delineating the placental microvessels and to explore the feasibility and potential clinical value of using MV-Flow for placental function in GDM women.

**Methods:**

This study included women with singleton pregnancies at 28–36 weeks of gestation and was conducted between November 2023 and January 2025. All women underwent routine prenatal ultrasound and MV-Flow. GDM women were stratified into two subgroups by glycated hemoglobin A_1c_ (HbA_1c_) value: GDM_1_ (HbA_1c_ ≤ 5.5%) and GDM_2_ (HbA_1c_ > 5.5%). Comparisons of data were made using parametric and non-parametric tests between the non-GDM group and the GDM group. Correlation between MVI_mean_ and neonate birthweight was assessed using Pearson’s correlation coefficient. Multivariate analysis was performed using general linear regression models of factors associated with GDM. Receiver operating characteristic curve analysis was conducted to determine the optimal MVI threshold for distinguishing between participants with GDM and those without.

**Results:**

The study population included 92 controls and 88 women with GDM. In the GDM group, compared to the control, there was significantly higher MVI_mean_ of placenta (P = 0.031). There was no significant difference between the GDM_2_ group and the control group in terms of MVI_mean_. There was a moderate correlation of placental MVI_mean_ with neonate birthweight (*r* = 0.539; *P* < 0.001). Based on the Youden index, the placental MVI_mean_ threshold that best discriminated between participants with GDM and those without was 38.95%, with a maximum achievable sensitivity of 62.9% and a specificity of 69.6%.

**Conclusions:**

Placental microvascular blood flow can be visualized and quantified in women with GDM using MV-Flow.

## Introduction

Gestational diabetes mellitus (GDM) is defined as an abnormal glucose tolerance first detected during pregnancy, and it affects approximately 14% of pregnancies annually worldwide (1). GDM is significantly associated with adverse pregnancy outcomes, including macrosomia, pre-eclampsia, fetal growth restriction, and preterm delivery (2). Glycated hemoglobin (HbA_1c_) reflects the average blood glucose over the most recent 2- to 3-month period (3). Although HbA_1c_ has limited utility as a diagnostic tool for GDM, it has been shown to be a reliable predictor of adverse outcomes associated with the condition (4, 5). According to the American Diabetes Association Professional Practice Committee, a target of HbA_1c <_6% is optimal during the third trimester (6). In a large cohort of women with GDM, Barbry et al. (7) found that a baseline of HbA_1c_ <5.6% predicted an increased risk of several adverse pregnancy outcomes.

The primary function of the placenta is to facilitate the exchange of substances between the mother and the fetus, ensuring the growth and development of the fetus. Mild to moderate placental dysfunction may impair the supply of nutrients and oxygen to the fetus, resulting in fetal distress and acquired brain damage, which may lead to lifelong diseases in the offspring (8). Previous studies demonstrated that GDM was associated with impaired placental development, showing villous immaturity or alterations in villous branching (9, 10). However, postnatal histopathological examination of the placenta cannot yield clinical biomarkers to inform the clinical management of pregnancy. It is uncertain whether placental perfusion changes can be observed *in utero* and whether these changes are influenced by the level of glycemic control in pregnant women.

At present, Doppler ultrasound forms the gold standard for monitoring placental insufficiency in clinical practice. Absent and reverse end-diastolic umbilical artery blood flow and the ductus venosus reversed a-wave indicate fetal death risk (11, 12). However, Doppler ultrasound is poorly sensitive to subtle changes in placental function and may not be altered until there are large disruptions. Previous studies have shown that microvascular flow imaging (MV-Flow) can display the stem villi and their branches and quantify placental microvascular structure (13, 14). The placental microvascular index (MVI) is a sensitive indicator of placental microcirculation (15). Chen et al. (16) found that the placental MVI in the group with congenital heart diseases and extracardiac malformations was significantly lower than that in the normal control group by MV-Flow. However, there is limited information about placental microvascular function in women with GDM. The placenta is a highly vascularized organ with branches of the umbilical artery and umbilical vein in the villi (17). Microvascular disease is a specific complication of diabetes mellitus (18, 19). The typical changes include microvascular basement membrane thickening and microcirculation dysfunction (20).

The objectives of this study were to accurately define placental microvascular function using MV-Flow in women with GDM and to compare these data to those obtained from women without GDM.

## Materials and methods

### Study population

A prospective cohort study design was utilized in this study. All pregnant women who participated in this study were recruited from November 2023 to January 2025 in our hospital and provided written or oral informed consent. This study was approved by the Ethics Committee of Obstetrics and Gynecology Hospital of Fudan University (Approval number: kyy2022-165). Singleton pregnancies with living fetuses and a gestational age (GA) of 28–36 weeks were identified in the cohort. The inclusion criteria were diagnosis of GDM confirmed by the oral glucose tolerance test (OGTT), age ≥18 years, and a pre-gestational body mass index (pre-BMI) <35 kg/m^2^. The inclusion criteria of the control group were as follows: 1) healthy women, 2) with euglycemia during pregnancy, and 3) with normal ultrasound scans and Doppler results. The common exclusion criteria for both groups were multiple pregnancies, major fetal abnormalities, abnormal karyotype, and pre-existing diabetes mellitus. A diagnosis of GDM was made at 24–28 weeks of gestation if the plasma glucose levels measured from the 75-g OGTT were met or exceeded in any of the following stages: 1) fasting: ≥ 5.1 mmol/L; 2) 1 h: ≥10.0 mmol/L; and 3) 2 h: ≥8.5 mmol/L (21). Women with GDM were categorized based on their HbA_1c_ values before delivery as GDM_1_ (HbA_1c_ ≤ 5.5%) or GDM_2_ (HbA_1c_ > 5.5%) (6, 7, 22).

### Maternal and fetal characteristics

We recorded information on maternal age, pre-BMI, pre-pregnant weight, pre-labor weight, gestational age at delivery, ultrasound scan, OGTT result, HbA_1c_ value before delivery, gravidity, parity, mode of delivery, neonate gender, Apgar score, and birthweight. At the clinic visit, we measured pre-pregnant and pre-labor weight and calculated gestational weight gain.

### Ultrasound imaging protocol

The prenatal ultrasound examinations were performed by an experienced sonographer transabdominally using the Samsung Hera W10 ultrasound systems (Samsung Medison Co., Gangwon-do, Korea) equipped with a curved transducer (2–9 MHz) and MV-Flow™ imaging technique. The name of the participant from the work list of the department was searched and checked, the date of the last menstrual period was entered, the appropriate obstetrical examination condition was set, and prenatal ultrasonography on the participant was performed according to the International Society of Ultrasound in Obstetrics and Gynecology (ISUOG) practice guidelines (23–26). Key metrics were recorded including placental thickness, maturity grade, umbilical artery (UmA) peak systolic velocity/end diastolic velocity (S/D), resistive index (RI), middle cerebral artery (MCA), pulsatility index (PI), RI, peak systolic velocity (PSV), uterine artery (UtA) PI, and UtA RI.

**Figure 1:**
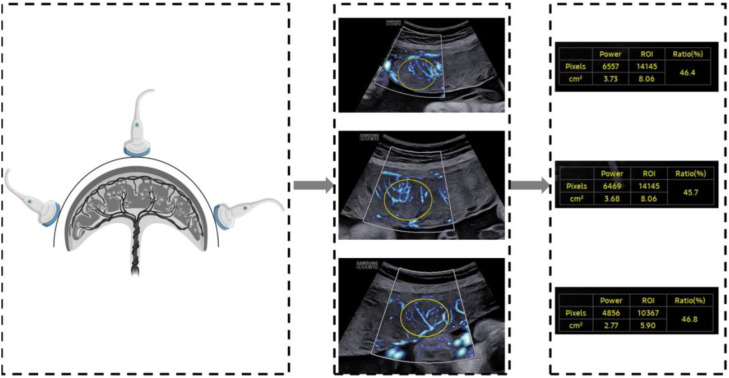
Demonstration of placental assessment using MV-Flow.

A two-dimensional transabdominal scan was performed to acquire a distinct image of the sagittal plane of the placenta. The instrument was set to the MV-Flow mode (frame avg = 8, dynamic range = 27, smooth = 1, filter = 3, sensitivity = 32, color map = 2), with the mechanical index and the thermal index adjusted to safe levels. The area of interest (ROI) was traced elliptically and displayed in square centimeters. Two-dimensional ultrasound images of the placental microvascular perfusion of the upper, middle, and lower sites were obtained, and the MVI values of the placenta were measured automatically (**Figure 1**). To represent the overall vessel microperfusion of the placenta, the mean MVI value (MVI_mean_) of the three sites was calculated for analysis. All placental MV-Flow analyses were performed by the study sonographer who underwent training with radiologist expertise in placental microvascular flow imaging and was blinded to maternal characteristics and GDM status. To assess the reproducibility of measurements, 40 cases from the control and GDM group were selected by stratified samplings, and measurements were taken by an expert investigator and then by the study sonographer, both of whom were blinded to previous measurements.

### Statistical analysis

Statistical analyses and graphs were conducted using SPSS 26.0 (IBM Corp., Armonk, NY, USA) and GraphPad Prism 9.5 (GraphPad Software, San Diego, CA, USA). Normally distributed continuous variables were presented as mean ± SD, and variables not following a normal distribution were presented as median (interquartile range, IQR). Nominal variables were summarized as number and percentage. The Student’s *t*-test or the Kruskal–Wallis test and the chi-square test were used to perform between-group comparisons of continuous and categorical variables, respectively, using *post hoc* SNK correction to adjust for multiple comparisons when necessary. Pearson correlation was used to evaluate the bivariate correlation between birthweight and MVI_mean_. General linear regression modeling was used to determine the associations between MVI_mean_ (%), pre-BMI (kg/m^2^), and pregnancy weight gain (kg) with birthweight (g). Standardized beta coefficients were estimated via multiple regression analysis to identify the variable most strongly associated with neonate birthweight. Independent variables did not show evidence of multicollinearity, with bivariate correlation coefficients <0.80. Intraclass correlation analysis was used to assess the intra- and interobserver variability of measuring placental MVI. Intraclass correlation coefficient (ICC) >0.70 was generally considered to be a good agreement. A *P*-value <0.05 was considered statistically significant.

## Results

### Baseline characteristics

A total of 88 women with GDM and 92 non-diabetic pregnant controls were included in the analysis. With the exception of 10 participants in the GDM group who lacked complete medical records, all the other participants had a complete dataset. Baseline characteristics and birth outcomes of the two groups are given in **Table 1**. Compared with controls, women with GDM were significantly older (*P* = 0.001), had a heavier pre-pregnant weight (59.89 ± 9.99 *vs*. 55.15 ± 6.06 kg, *P* < 0.001), had a higher pre-BMI (22.77 ± 3.42 *vs*. 20.92 ± 2.31 kg/m^2^, *P* < 0.001), and had a thinner gestational weight gain [10.9 (IQR, 7.05–15.15) *vs*. 14 (IQR, 10.5–16.39) kg, *P* < 0.001]. The gestational age at the time of a scan was later in the GDM group [32.65 (IQR, 30.3–34.68) *vs*. 29.6 (IQR, 28.9–33.7) weeks, *P* < 0.001). Among the GDM participants, 20.51% were on insulin alone, 7.69% were on insulin and metformin in combination, and 71.79% were on dietary management. Four cases followed spontaneous labor with intact membranes, and one case followed preterm premature rupture of the membranes. GDM pregnancies were associated with a significantly increased rate of preterm delivery (6.41% vs. 0%, *P* = 0.044), but insignificantly reduced final birthweight [3,210 (IQR, 3,035–3,425) *vs*. 3,285 (IQR, 3,085–3,492.5) g, *P* = 0.153]. No significant differences were observed between the two groups with respect to maternal height, prevalence of maternal hypertensive disorders of pregnancy, gestational age at delivery, rate of male neonate, neonatal NICU admission, respiratory distress, or hyperglycemia. Of note, prothrombin time (*P* = 0.392), activated partial thromboplastin time (*P* = 0.372), fibrinogen (*P* = 0.315), thrombin time (*P* = 0.836), and D-dimer (*P* = 0.13) did not differ significantly between the two groups.

**Table 1:** Demographic characteristics of the participants included in the study.

Characteristics	Non-GDM (*n* = 92)	GDM (*n* = 88)	*P*
Age (years)	30.5 (28, 33)	32 (30, 35)	0.001*
Height (m)	1.63 ± 0.05	1.62 ± 0.05	0.514
Pre-pregnant weight (kg)	55.15 ± 6.06	59.89 ± 9.99	<0.001*
Pre-BMI (kg/m^2^)	20.92 ± 2.31	22.77 ± 3.42	<0.001*
Gestational weight gain (kg)	14 (10.5, 16.39)	10.9 (7.05, 15.15)	<0.001*
GA at scan (weeks)	29.6 (28.9, 33.7)	32.65 (30.3, 34.68)	<0.001*
Parity			0.033*
Parous			
Previous GDM	0 (0.0)	1 (1.28)	
No previous GDM	15 (16.30)	23 (29.49)	
Nulliparous	77 (83.70)	54 (69.23)	
Treatment for GDM			
Diet	–	56 (71.79)	
Insulin	–	16 (20.51)	
Insulin with metformin	–	6 (7.69)	
Medical history			
HDP	5 (5.43)	11 (14.10)	0.054
SLE/APS	0 (0.0)	2 (2.56)	0.209
GA at delivery (weeks)	39.6 (38.7, 40.25)	39.2 (38.4, 39.9)	0.053
Mode of delivery			0.183
Vaginal birth	61 (66.30)	59 (75.64)	
Cesarean section	31 (33.70)	19 (24.36)	
HbA_1c_	4.8 (4.6, 5)	5.2 (5, 5.4)	<0.001*
BW (g)	3,285 (3,085, 3,492.5)	3,210 (3,035, 3,425)	0.153
BW category			0.082
SGA	0 (0.0)	3 (3.85)	
AGA	91 (98.9)	72 (92.30)	
LGA	1 (1.1)	3 (3.85)	
Male neonatal sex	47 (51.1)	45 (57.69)	0.389
Preterm birth	0 (0.0)	5 (6.41)	0.044*
1-min Apgar score <7	0 (0.0)	3 (3.84)	0.189
Acidosis (pH ≤7.0 or lactate >6)	0 (0.0)	2 (2.56)	0.209
NICU admission	0 (0.0)	1 (1.28)	0.459
Respiratory distress	0 (0.0)	2 (2.56)	0.209
Hyperglycemia	0 (0.0)	2 (2.56)	0.209

Data are shown as mean ± SD, median (interquartile range), or *n* (%). Comparisons between groups were made using chi-square or Fisher’s exact test for categorical variables and Student’s *t*-test or Mann–Whitney *U*-test for continuous variables.

GA, gestational age; BMI, body mass index; HDP, hypertensive disorders of pregnancy; SLE, systemic lupus erythematosus; APS, antiphospholipid syndrome; BW, birthweight; SGA, small for gestational age; AGA, appropriate for gestational age; LGA, large for gestational age; NICU, neonatal intensive care unit.

*P < 0.05.

### Ultrasound assessment at 28–36 weeks of gestation

**Figure 2:**
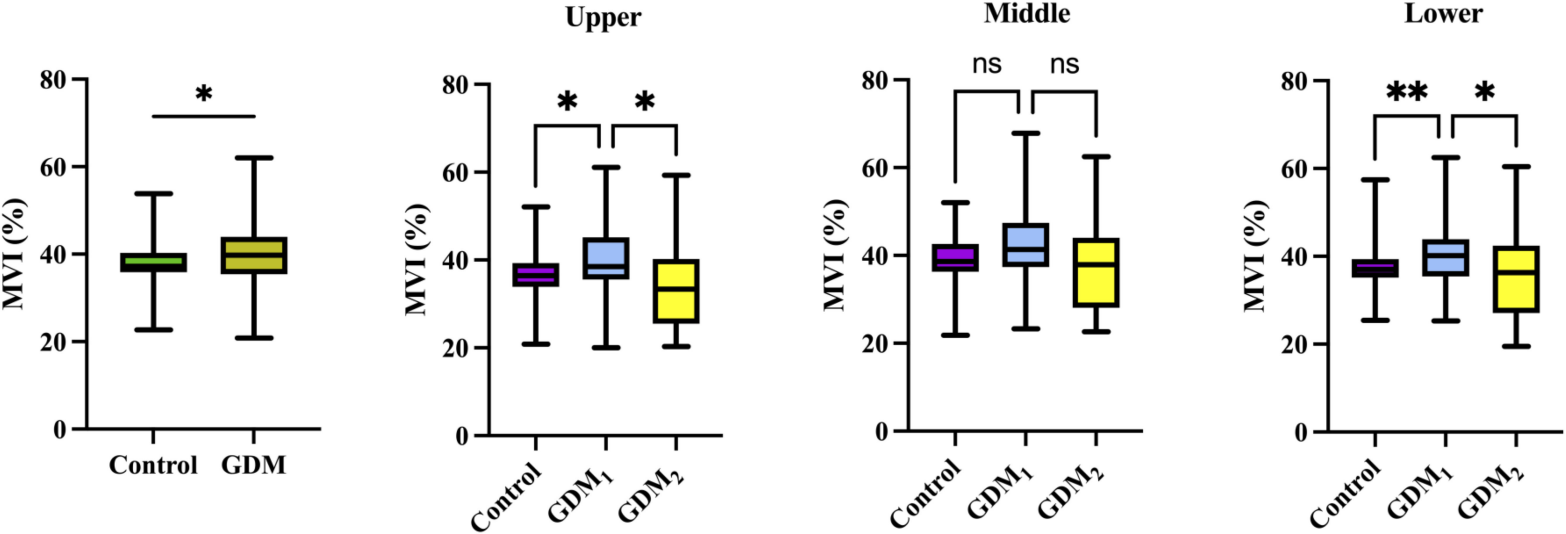
Box plots of MVI values in the control (*n* = 92), GDM (*n* = 88), GDM_1_ (*n* = 70), and GDM_2_ (*n* = 18) groups. Statistics was performed by the Mann–Whitney or Kruskal–Wallis test. ^**^P < 0.01; ^*^P < 0.05; ^ns^*P* > 0.05.

Ultrasound measurements for participants with GDM or uncomplicated pregnancies are summarized in **Table 2**. There was no significant difference in the prevalence of bilateral notch uterine artery at 11–14 weeks between the control and GDM_1_ group, but the GDM_2_ group had higher values compared to the other two groups. Women in the GDM_2_ group, compared with those in the GDM_1_ group, had greater MCA PI during pregnancy. Compared with controls, women with GDM had significantly higher placental MVI_mean_ (P = 0.031, **Figure 2**). The placental MVI_mean_ of the control or GDM_2_ group was significantly lower than that of the GDM_1_ group (all *P* < 0.05). **Figure 2** also shows group comparisons between the control, the GDM1 group, and the GDM2 group regarding each of the three MV-Flow parameters obtained in the placenta. Participants in the GDM1 group had greater MVI values in the upper or lower parts of the placenta than participants in the control and GDM2 group (all P < 0.05). None of the MVI in the other parts of the placenta showed group differences. There was no significant difference in mean UtA PI, mean UtA RI, UmA PI, UmA RI, UmA S/D, MCA RI, MCA PSV, cerebroplacental ratio, placental thickness, and maturity grading between the groups.

**Table 2:** Ultrasound parameters.

Variable	Group			*P*
	Control (*n* = 92)	GDM_1_ (*n* = 70)	GDM_2_ (*n* = 18)	
Mean UtA PI	1.65 ± 0.55	1.61 ± 0.49	1.82 ± 0.52	0.372
Mean UtA RI	0.73 (0.66–0.82)	0.74 (0.66–0.79)	0.75 (0.72–0.82)	0.308
Bilateral notch UtA	13 (14.13%)	7 (12.96%)	8 (53.33%)^ab^	0.003^*^
UmA PI	0.96 ± 0.16	0.97 ± 0.15	1 ± 0.19	0.489
UmA RI	0.63 (0.58–0.66)	0.64 (0.59–0.66)	0.64 (0.59–0.71)	0.593
UmA S/D	2.68 (2.39–2.94)	2.69 (2.45–2.91)	2.79 (2.42–3.5)	0.379
MCA PI	2.06 ± 0.4	1.89 ± 0.29	2.19 ± 0.36^b^	0.006^*^
MCA RI	0.86 (0.81–0.9)	0.84 (0.81–0.88)	0.88 (0.86–0.9)	0.132
MCA PSV (cm/s)	40.88 ± 6.84	39.08 ± 8.12	40.8 ± 9.64	0.345
Cerebroplacental ratio	2.07 (1.81–2.54)	1.93 (1.73–2.32)	2.29 (1.99–2.53)	0.076
Placental parameters				
Thickness (mm)	31.37 ± 4.27	33.17 ± 4.44	32.65 ± 3.71	0.211
Maturity grading	2 (1–2)	2 (1–2)	2 (1–2)	0.456
MVI_mean_ (%)	37.25 (35.93–40.28)	40.35 (36.38–45.08)^a^	35.95 (26.75–42.1)^b^	0.004^*^

Data are given as mean ± SD, median (range), or *n* (%).

UtA, uterine artery; UmA, umbilical artery; MCA, middle cerebral artery; PI, pulsatility index; RI, resistive index; PSV, peak systolic velocity; S/D, peak systolic velocity/end diastolic velocity; MVI_mean_, mean microvascular index.

a *P* < 0.05 vs. control.

b *P* < 0.05 vs. GDM_1_; ^*^P < 0.05.

**Figure 3:**
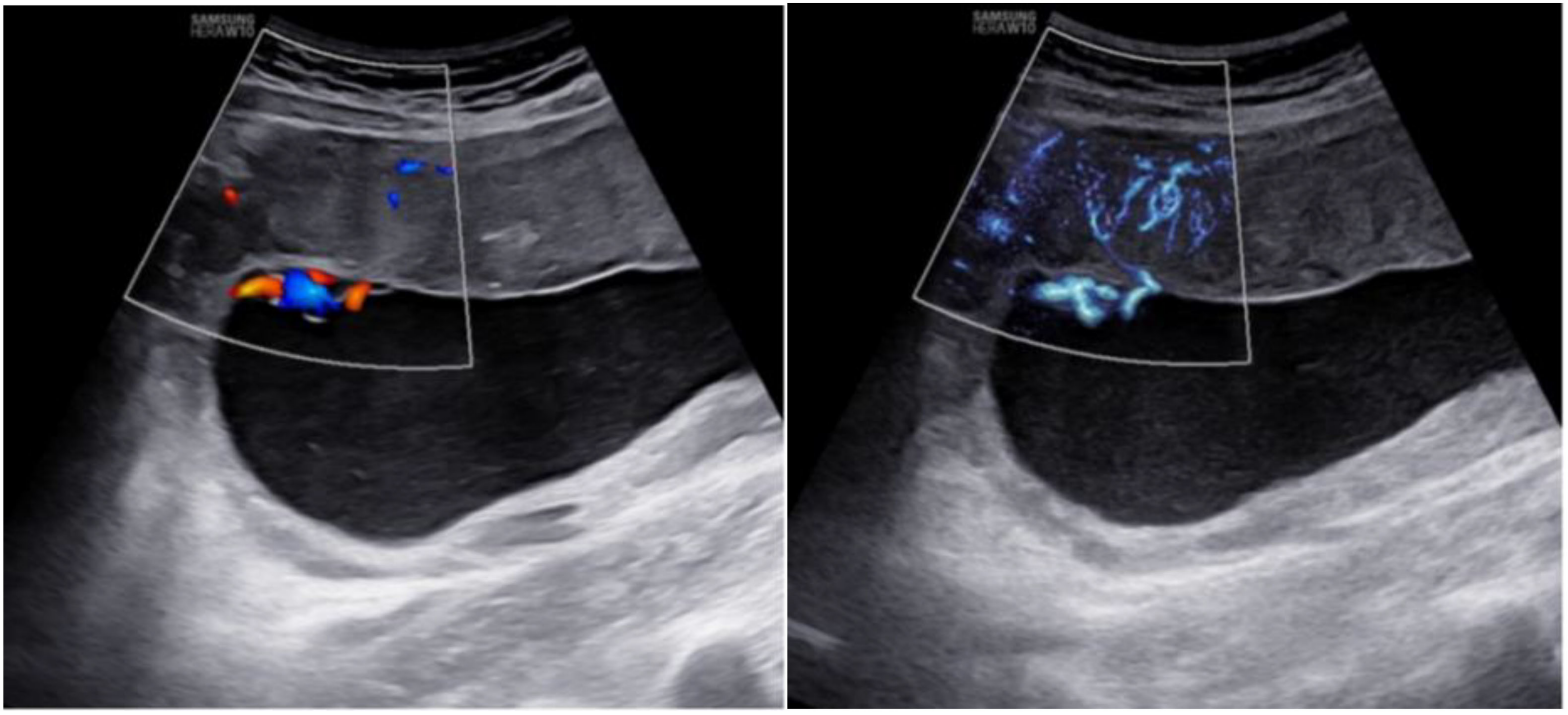
Placental imaging by routine Doppler ultrasound (left) and MV-Flow (right).

**Figure 4:**
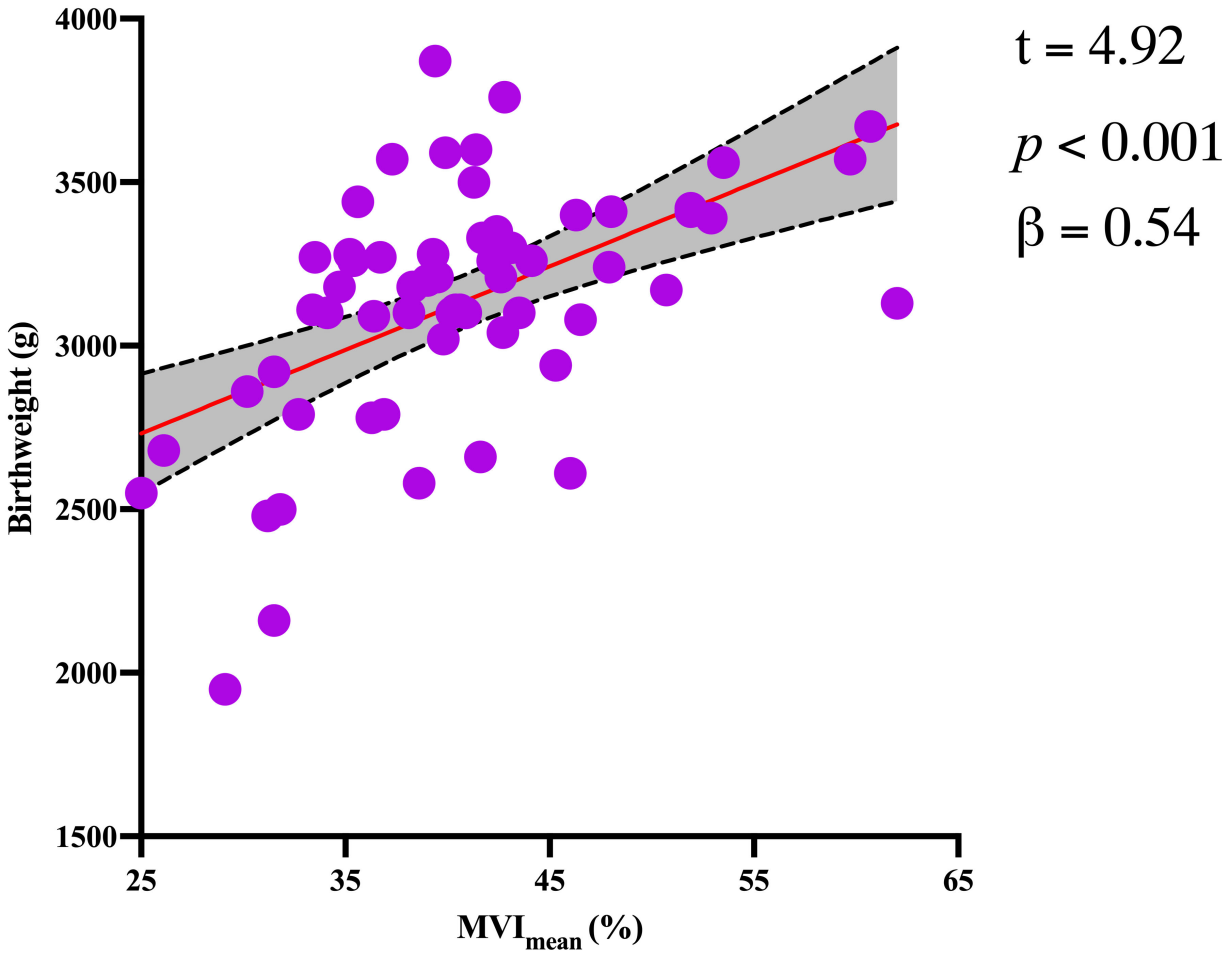
Significant association between birthweight and MVI_mean_ for the GDM_1_ group. The dotted lines represent the 95% confidence interval.

**Figure 5:**
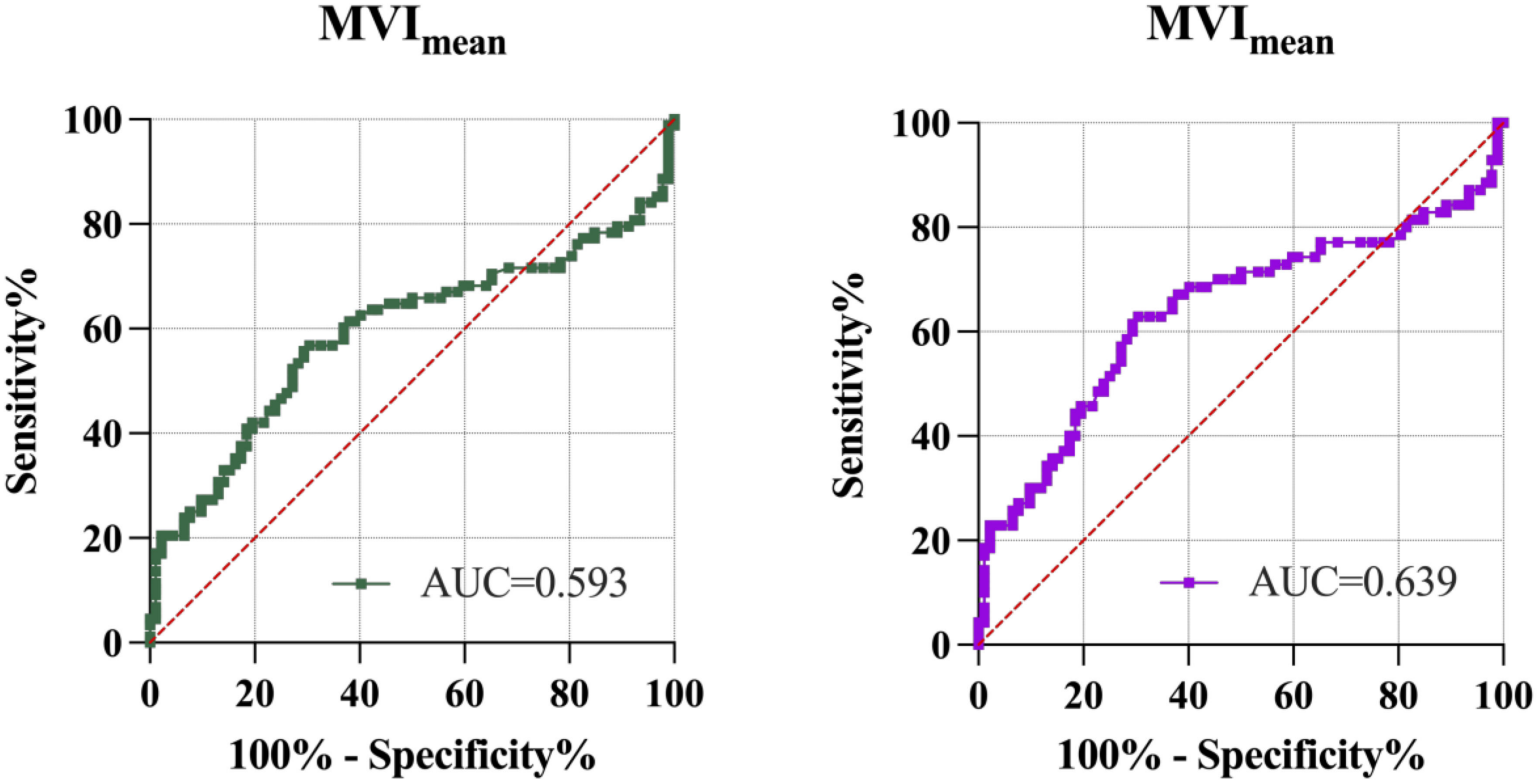
ROC analysis of MVI_mean_ to discriminate normal and GDM pregnancies. Left figure, control group and the GDM group. Right figure, control group and the GDM_1_ group. AUC, area under the curve; MVI_mean_, mean microvascular index.

In addition, MV-Flow imaging was able to depict smaller, slow-flow vessels within the placenta, which were not visible using routine Doppler ultrasound (**Figure 3**). **Figure 2** shows group comparisons between the control, the GDM_1_ group, and the GDM_2_ group regarding each of the three MV-Flow parameters obtained in the placenta. Participants in the GDM_1_ group had greater MVI values in the upper or lower parts of the placenta than participants in the control and GDM_2_ group (all *P* < 0.05). None of the MVI in the other parts of the placenta showed group differences. There was a moderate correlation between MVI_mean_ (%) and birthweight (g) in the GDM_1_ group (Pearson: *r* = 0.539; *P* < 0.001). However, there was no significant correlation between MVI_mean_ (%) and birthweight (g) in the GDM_2_ group (Pearson: *r* = −0.416; *P* = 0.109). In the secondary analyses within GDM_1_, higher MVI_mean_ (40.81% ± 7.83%) was significantly associated with higher neonate birthweight (3,135.41 ± 370.64 g) (adj.*R*^2^ = 0.28, *F* = 24.22, *P* < 0.001; *t* = 4.92, *P* < 0.001; standardized *β* = 0.539) (**Figure 4**). Pregnancy weight gain, pre-BMI, MVI_mean_, and birthweight were included in the multivariable analysis. MVI_mean_, pregnancy weight gain, and pre-BMI were positively associated with birthweight (standardized *β* = 0.532, *P* < 0.001; standardized *β* = 0.236, *P* = 0.027; standardized *β* = 0.307, *P* = 0.004, respectively). The receiver operating characteristic (ROC) curve analysis indicated that MVI_mean_ can well discriminate the normal and GDM pregnancies, with an area under the curve (AUC) value of 0.593 ([95% CI, 0.507–0.68]; *P* = 0.031; **Figure 5**). The sensitivity and specificity of MVI_mean_ for discriminating between normal and GDM pregnancies were 56.8% and 69.6%, respectively. After the GDM_2_ group was excluded from the GDM group, the results suggested that MVI_mean_ was able to separate well GDM pregnancies from normal pregnancies, with an AUC value of 0.639 ([95% CI, 0.547–0.731]; *P* = 0.003; **Figure 5**). The Youden index, indicating the optimal point along the ROC curve for GDM prediction, was calculated at the MVI_mean_ level of 38.95%, with a specificity of 69.6% and sensitivity of 62.9% at this level.

### Repeatability test

The placental MVI_mean_ measurements were performed twice on placentas from 20 women in the control group, 15 women in the GDM_1_ group, and 5 women in the GDM_2_ group by the study sonographer. The consistency of the study sonographer’s measurements was good. The ICC values for the study sonographer’s measurements (conducted twice) were 0.80, 0.94, and 0.84, respectively. The consistency between the values of the two doctors’ measurements was good, with ICC values of 0.90, 0.93, and 0.72, respectively.

## Discussion

### Main findings

To our knowledge, this is the first study to evaluate the association between placental MVI_mean_ and neonate birthweight in participants with GDM. Our main findings were that placental MVI_mean_ were moderately associated with neonate birthweight in participants who had GDM with good glycemic control. Of the variables evaluated in this paper, placental MVI_mean_ was found to be most strongly associated with neonate birthweight. In this study, we demonstrated that women with GDM, compared to those without GDM, showed an increase in placental function. There were no significant differences between the normal pregnancies and the GDM group with poor glycemic control in placental MVI_mean_ assessed by MV-Flow. These data suggest that cerebroplacental ratio, placental thickness, and maturity grading have not been affected within the development of GDM so far and that MV-Flow imaging is a sensitive ultrasonic Doppler imaging technology to assess early placental functional changes in women with GDM.

### Interpretation

Our work in GDM women demonstrated that the placental MVI_mean_ initially increased and subsequently decreased when comparing the GDM_1_ group to the GDM_2_ group, which was partly consistent with a previous study (27). However, the previous study did not include GDM women with poor glycemic control. Driven by maternal hyperglycemia and fetal hyperinsulinemia in pregnancies complicated by GDM, fetal metabolic demands increase significantly, resulting in a relatively placental hypoxic environment (28). This condition activates key signaling pathways, including HIF-1α and VEGF, thereby triggering the placental angiogenic response (29). This initial response is evident on MV-Flow imaging as elevated placental MVI. Continuous high-glucose environments can activate the glycolysis pathway and induce excessive reactive oxygen species production, increasing oxidative stress, which causes mitochondrial defects, cellular apoptosis, and inflammation (30). Placentas from women with GDM have decreased regulatory T cells and increased NK cells, neutrophil infiltration and activation, and macrophage activation (31). Significantly upregulated pathways in GDM placentas included several immune responses (IL-1β, IL-2, IL-6, TNFα, IFNγ, TGF-β) and downregulation of glycolysis and cell cycle pathways (31–33). This pro-inflammatory and pro-oxidative environment promotes the upregulation of sFlt-1 (anti-angiogenic factor) while simultaneously reducing the reactivity of vascular endothelial cells to VEGF and PlGF (pro-angiogenic factors), thereby interfering with the balance of angiogenic factors (34, 35). However, this vascular proliferation represents a spectrum of adaptation—from successful “adaptive compensation” to failed “pathological decompensation.” The ultimate impact on pregnancy outcomes depends on the structural and functional integrity of the newly formed blood vessels (36). Studies of gross morphology and histoarchitecture in placentas delivered from GDM pregnancies consistently reported increased placental size, weight, thickness, chronic parenchymal inflammation, villous immaturity, and vascular thrombosis when compared to uncomplicated pregnancies (37–39).

To assess the whole placental status of our participants, we also used the Doppler ultrasound technique, which provides information on UtA and UmA hemodynamics. Our results demonstrated no statistically significant differences in the hemodynamic parameters (PI, RI, and S/D) of the UtA and UmA across the three groups. Prior literature has observed UmA Doppler deterioration in severe cases with placental vascular dysfunction (40–42). This means the results of umbilical artery Doppler imaging may be a late marker of placental dysfunction. MV-Flow can detect early changes in placental microvascular density, representing a key advantage over traditional Doppler techniques. It can also offer a clearer display of villi and a greater sensitivity to low flow. In addition, our analysis found that fetal MCA-PI in GDM_2_ pregnancies is higher than in GDM_1_ pregnancies, indicating a greater degree of fetal hypoxia, which is consistent with a previous study (43).

A well-developed placental microvascular network provides a more efficient interface for maternal–fetal exchange and ensures a more robust blood supply to support fetal development. This directly enhances the fetal uptake of essential nutrients, including glucose, amino acids, and fatty acids, thereby supporting normal and, in some cases, accelerated growth (44, 45). Our finding of a positive relationship between MVI_mean_ and birthweight in the GDM_1_ group, which is approximately in line with a previous work using a large-scale sample, shows that the placental–fetal growth nexus is reflected by a positive correlation (*r* = 0.6) between the placenta and birthweight (46). Therefore, this discovery provides direct imaging evidence that placental microvascular network serves as an important factor of fetal weight. In the subgroup with poor glycemic control, the association between MVI_mean_ and birthweight was attenuated or no longer evident, potentially attributable to microvascular dysfunction. This MVI_mean_ cannot represent the efficiency of material exchange in the placental microcirculation. Prolonged exposure of the fetus to a hyperglycemia-induced hyperinsulinemic environment resulting from maternal hyperglycemia enhances protein and fat synthesis, thereby promoting excessive fetal growth and increasing the risk of macrosomia (47). Chronic hyperglycemia may result in dysfunction of villous vascular endothelial cells, thickening of the vascular basement membrane, and even microthrombosis (48). The presence of both 6.25% small-for-gestational-age and 18.75% large-for-gestational-age infants in the poor glycemic control group was observed, which is consistent with the aforementioned finding.

Our study investigating the diagnostication of GDM_1_ based on the placental MVI_mean_ alone in the third trimester reported an AUC of 0.639. Its clinical utility and diagnostic power are fundamentally limited. MV-Flow may capture a component of the placental pathophysiological changes associated with GDM but lacks the discriminatory strength to serve as a robust standalone diagnostic or screening tool. The limited diagnostic accuracy of MV-Flow may be attributed to inherent limitations in its technical principles as well as a high degree of operator dependency (49). MV-Flow is designed to visualize low-velocity blood flow; however, this capability may increase susceptibility to motion artifacts and could be constrained by the probe’s penetration depth and spatial resolution. Based on the above limitations, we believe that the role of MV-Flow in clinical practice should be redefined as an exploratory and auxiliary tool. Its main value lies in complementing rather than replacing traditional color Doppler ultrasound and other mature imaging methods (such as contrast-enhanced ultrasound or magnetic resonance imaging). We believe that it is more accurate to position placental MVI_mean_ as a functional imaging biomarker, which has both prognostic and monitoring potential, but it is not yet suitable for independent diagnosis. There are conflicting reports of the impact of GDM on sFLt-1, PlGF, and its ratio. While Pankiewicz et al. (50) and Noonan et al. (51) found no significant difference in the sFlt-1/PlGF ratio when comparing individuals with GDM and pre-eclampsia (PE) and individuals with PE, Nuzzo et al. (34) found that the sFlt-1/PlGF ratio was significantly lower in individuals with GDM-PE than individuals with PE. Gibbons et al. (43) reported that low cerebroplacental ratio was associated with poorer neonatal outcome in women with GDM. However, Cardinali et al. (52) showed that cerebroplacental ratio is associated but not predictive of adverse perinatal outcome in pregnancies complicated by gestational diabetes. Future research should focus on integrating MVI_mean_ with other clinical, biochemical, or sonographic markers within a multivariate model to determine if it can provide incremental value in improving overall predictive performance.

### Strengths and limitations

To our knowledge, this is the first study to investigate placental microvascular function in GDM throughout the third trimester rather than during different gestational age. We used a non-invasive and reproducible technique, which has been shown to offer information for placental microvascular network and perfusion in GDM women. We also considered women with GDM on different levels of glycemic control separately and as an independent group, allowing more detailed characterization of placental microvascular architecture in pregnancies complicated by GDM compared to routine color Doppler ultrasound. In addition, placental MVI measurements are technically feasible in a busy public clinic.

The main limitations of this study are that our population was primarily Asians. It is known that different races/ethnic groups vary in body composition, insulin sensitivity, susceptibility to diabetes, and the risk of pregnancy-related complications. Thus, our results might not be applicable to women of other racial origins. Second, superb microvascular imaging measurements may vary when equipment from different vendors is used for analysis. This variability should be taken into account when comparing results across studies, and efforts should be made to standardize measurement protocols across platforms. Third, limitations of our study include the absence of histological confirmation of placenta. Furthermore, we acknowledge that a formal sample size calculation was not performed prior to the study. The study utilized a convenience sample of 180 (control:GDM_1_:GDM_2_ = 92:70:18) participants, which was constrained by successful clinical management of GDM at present. Consequently, further investigations involving larger, multi-ethnic, and multicenter cohorts are needed to validate and substantiate the clinical value of placental MVI measurement in identifying placental dysfunction accurately.

## Conclusion

In women with GDM, there are subtle placental functional changes, and these can be detected using MV-Flow. Compared with traditional color Doppler ultrasound, the advantages of placental microvascular imaging are revolutionary, achieving a leap from assessing “macroscopic blood flow” to displaying “microscopic structure.” The microvascular scans of the placenta were demonstrated approaching real time, which enabled measurements and rendering of placental villus structure features. MV-Flow may help supplement traditional color Doppler ultrasound and magnetic resonance imaging in diagnosing and monitoring various placenta-related pregnancy diseases. Further studies are needed to describe the placental pathology alterations in women with GDM and to verify our prenatal findings.

## Data Availability

The original contributions presented in the study are included in the article/supplementary material. Further inquiries can be directed to the corresponding author.
